# Effects of Peroxyl Radicals on the Structural Characteristics and Fatty Acid Composition of High-Density Lipoprotein from Duck Egg Yolk

**DOI:** 10.3390/foods11111634

**Published:** 2022-06-01

**Authors:** Jing Sun, Qi Zeng, Xue Yang, Jinsong Pi, Meihu Ma, Jinping Du

**Affiliations:** 1 College of Food Science and Technology, Huazhong Agricultural University, Wuhan 430070, China; sammi8866@sina.com (J.S.); qizeng0310@163.com (Q.Z.); 2 Institute of Animal Husbandry and Veterinary, Hubei Academy of Agricultural Science, Wuhan 430072, China; yangxue15826@sina.com (X.Y.); pijinsong@sina.com (J.P.); 3 Hubei Key Laboratory of Animal Embryo and Molecular Breeding, Hubei Academy of Agricultural Science, Wuhan 430072, China

**Keywords:** egg yolk, high-density lipoprotein, peroxyl radicals, oxidation, fatty acid composition

## Abstract

In this study, high-density lipoprotein (HDL) from duck egg yolk was subjected to oxidation with a system based on 2,2′-azobis (2-amidinopropane) dihydrochloride (AAPH)-derived peroxyl radicals. The effects of peroxyl radicals on the protein carbonyl, free sulfhydryl, secondary/tertiary structure, surface hydrophobicity, solubility, particle size distribution, zeta potential and fatty acid composition of HDL were investigated by using sodium dodecyl sulfate polyacrylamide gel electrophoresis (SDS-PAGE), Fourier-transform infrared spectroscopy (FTIR), circular dichroism (CD), fluorescence spectroscopy, dynamic light scattering and ultra-high-performance liquid chromatography coupled to tandem mass spectrometry (UHPLC-MS/MS). The results indicated that the content of protein carbonyl was significantly increased, that of free sulfhydryl was obviously reduced, and the ordered secondary structure was also decreased with increasing AAPH concentration. In addition, the surface hydrophobicity and solubility of HDL showed apparent increases due to the exposure of hydrophobic groups and aggregation of protein caused by oxidation. The fatty acid composition of HDL exhibited pronounced changes due to the disrupted protein–lipid interaction and lipid oxidation by AAPH-derived peroxyl radicals. These results may help to elucidate the molecular mechanism for the effect of lipid oxidation products on the oxidation of duck yolk proteins.

## 1. Introduction

Egg yolk (EY) is constituted by 80% plasma and 20% water-insoluble granules. Besides, it consists of 32% lipids and 16% proteins, mainly including phosvitin (PV), immunoglobulin Y (IgY), low-density lipoprotein (LDL), high-density lipoprotein (HDL) and lecithin [1]. EY-HDL is a globule-like pseudo-molecular structure that binds to PV through calcium phosphate bridges, forming a complex yolk granule structure containing embedded EY-LDL vesicles [2]. It is a spherical molecule with a molecular weight of about 400 kDa and consists of four subunits, namely two lipovitellin-1 (~125 kDa) and two lipovitellin-2 (~25 kDa) in terms of protein structure [3,4]. The lipid part accounts for about 25% of the total molecular weight and mainly includes phospholipids, triglycerides and cholesterol [5]. The interaction forces between lipids and proteins in HDL are mainly hydrophobic bond and electrostatic force. A funnel-shaped cavity with a volume of about 68 nm^3^ is formed inside HDL apolipoglobulin by the β-sheet of two hydrophobic amino acids. This cavity is large and can accommodate up to 35 phospholipid molecules. Phospholipids interact with hydrophobic amino acids on the monolayer, while triglycerides can be further encapsulated in this hydrophobic cavity by new hydrophobic domains established by phospholipids [6,7]. There are also large amounts of salt and ion bridges inside the cavity, which together maintain the stability of the cavity-wrapping structure. Electrostatic interaction also plays an important role in maintaining the HDL structure. Previous studies have shown that, in a 1 mol/L NaCl solution, the decomposition rate of HDL was greatly reduced, while sulfhydryl and protein phosphorus groups were not directly involved in the decomposition reaction, indicating that the stability of the HDL dimer structure is dependent on the joint effect of electrostatic force and hydrophobicity [8,9].

The lipid oxidative products (such as cholesterol, phospholipid and fatty acid oxidative products) in egg yolk have attracted increasing attention. It has been reported that these oxidative products have negative effects on the gel and emulsifying properties of egg yolk, which may lead to the occurrence of coronary artery disease and some cancers after intake [10,11]. However, such effects may be prevented by some antioxidant compounds [12]. The oxidative products from fatty acids are mostly hydroperoxides, which are extremely unstable primary oxidative products and can be degraded to produce various decomposition products [13,14]. Hydroperoxides can generate alkoxy radical RO upon the breaking of their oxygen–oxygen bonds or other compounds such as aldehydes, acids and hydrocarbons upon the breaking of their carbon–carbon bonds. In addition, RO can also be decomposed into ketones, alcohols and aldehydes, such as the secondary oxidation products malondialdehyde (MDA), α, β-unsaturated aldehyde and hexanal [15,16,17].

Protein oxidation has been demonstrated to have significant impacts on the quality of protein-rich foods [18,19]. During the processing and storage of egg products, egg yolk is easily oxidized to produce some hydroperoxides and radicals, which thereby induce the oxidation of proteins and affect their structural characteristics and physicochemical properties, resulting in the deterioration of egg products [20]. 2,2′-Azobis (2-amidinopropane) dihydrochloride (AAPH) can be thermally decomposed at 37 °C in the dark to produce peroxyl radicals. Therefore, it can be used as an effective peroxyl radical producer to investigate the oxidation process of proteins [21,22]. In this study, HDL from duck egg yolk was exposed to different concentrations of peroxyl radicals generated with AAPH, aiming to explore the effects of peroxyl radicals on the protein carbonyl, free sulfhydryl, secondary/tertiary structure, surface hydrophobicity, solubility, particle size distribution, zeta potential and fatty acid composition of HDL.

## 2. Material and Methods

### 2.1. Materials and Reagents

Duck eggs were obtained from Charoen Pokphand Egg Industry Co. Ltd. (Wuhan, China). 2,2′-Azobis (2-amidinopropane) dihydrochloride (AAPH), 2,4-dinitrophenylhydrazine (DNPH), 5,5′-dithiobis(2-nitrobenzoic acid) (DTNB) and 8-anilino-1-naphthalenesulfonic acid (ANS) were purchased from Shanghai Macklin Biochemical Technology Co., Ltd. (Shanghai, China). SDS-PAGE kits were purchased from Beijing Lanjieke Technology Co., Ltd. (Beijing, China). Trichloroacetic acid, guanidine hydrochloride, ethyl acetate and other chemicals of analytical grade were purchased from Sinopharm Co. (Beijing, China). All fatty acid standards and stable isotope-labeled standards were obtained from ZZ Standards Co., Ltd. (Shanghai, China). Isopropanol (Optima LC-MS), acetonitrile (Optima LC-MS) and formic acid (Optima LC-MS) were purchased from Thermo-Fisher Scientific (Fair Lawn, NJ, USA). Ultrapure water was purchased from Millipore (Boston, MA, USA).

### 2.2. Preparation of High-Density Lipoprotein (HDL) from Duck Egg Yolk

Duck egg yolk was collected using an injector after the complete separation of egg white and yolk. The collected sample was mixed with 0.15 M NaCl solution at a volume ratio of 5:1 and incubated at 4 °C for 12 h, followed by centrifugation at 8000× *g* for 30 min. Then, the precipitate was collected and dissolved in 0.15 M NaCl solution to reach a protein concentration of 10%. Next, the pH of the solution was adjusted to 3.7 prior to centrifugation at 8000× *g* for 30 min [7]. Finally, the precipitate was collected and then lyophilized to obtain high-density lipoprotein (HDL) powder.

### 2.3. Preparation of Oxidized HDL

HDL solution at 10 mg/mL was prepared, followed by adjustment of the pH to 8.0 to promote the dissolution of HDL. AAPH at different concentrations (0.05, 0.25, 1.25, 6.25, 12.5 mM) was added into HDL solution and the mixture was incubated at 37 °C for 24 h, followed by desalination using a dialysis bag [23]. The oxidized HDL powder was obtained from the resultant solution by lyophilization.

### 2.4. SDS-PAGE Analysis

Oxidized HDL solution (2 mg/mL) was mixed with loading buffer at a volume ratio of 4:1, followed by a boiling water bath for 5 min. Separating and stacking gels were prepared using SDS-PAGE kits, and the specific operations were carried out according to the instructions. In this experiment, the concentration of the separating gel was 12% and that of the stacking gel was 8%. About 15 μL of sample was loaded, and the molecular weight range of the pre-stained protein marker was 10–250 kDa. The stacking gel voltage was 80 V, and the separating gel voltage was 120 V. After electrophoresis, the gel was first fixed in fixative solution for 40 min and then transferred to a staining solution for 40 min. Finally, the gel was de-stained in a de-staining solution, which was replaced during the process until the disappearance of the background color [24].

### 2.5. Measurements of Protein Carbonyl and Free Sulfhydryl

The carbonyl content in oxidized HDL was determined by reacting the carbonyl group with 2,4-dinitrophenylhydrazine (DNPH) under acidic conditions [25]. About 1 mL of protein solution (5 mg/mL, determined by Coomassie brilliant blue method) and 1 mL of 10 mmol/L DNPH solution were mixed in a centrifuge tube and placed in the dark at room temperature for 1 h (shaken every 15 min). Then, 3 mL of 20% trichloroacetic acid was mixed with the mixture and centrifuged at 4 °C and 10,000× *g* for 5 min. The supernatant was removed, and the precipitate was washed with ethyl acetate/ethanol (*v*/*v*, 1:1). The precipitate was then dissolved in 5 mL of 6 mol/L guanidine hydrochloride solution and incubated at 37 °C for 30 min in a water bath. After that, the resultant solution was centrifuged at 10,000× *g* for 10 min. The absorbance of the supernatant was measured at 370 nm.

Ellman’s reagent was prepared by dissolving 4 mg of 5,5′-dithiobis (2-nitrobenzoic acid) (DTNB) in 1 mL of Tris-glycine buffer (pH 8.0). Subsequently, 1 mL of Tris-glycine buffer with 8 mol/L urea was mixed with 1 mL of 2 mg/mL oxidized HDL solution. Then, 50 μL of Ellman’s reagent was added into the mixture, which was incubated at room temperature for 30 min. After that, the reaction solution was centrifuged at 8000× *g* for 20 min, and the absorbance of the samples was measured at a wavelength of 412 nm [26]. The free sulfhydryl content was calculated according to the following formula (1).

(1) $$Free\;SH\;(\mu mol/g\;protein)=75.53\;\times \;A_{412}\;\times \;D/C\;$$

where *A*_412_ is the absorbance of samples, *D* is the dilution factor and *C* is the protein concentration (mg/mL).

### 2.6. FTIR and CD Analysis

Oxidized HDL powder was analyzed by infrared spectroscopy (Thermo Nicolet Nexus 470 infrared spectrometer with DTGS detector) using the KBr tablet method (sample mixed with KBr at 1:100). The scanning parameters were as follows: wavenumber range, 400–4000 cm^−1^; resolution, 4.0 cm^−1^; and scan times, 32. OMNIC 8.2 (Thermo Nicolet Company) software was used to acquire the infrared spectra and analyze the characteristic peaks.

Oxidized HDL powder was dissolved in distilled water to prepare a 0.5 mg/mL protein solution. The CD spectra were collected in the far ultraviolet region (190–260 nm), with a colorimetric cell diameter of 0.1 cm, a spectral band width of 1.0 nm, a sensitivity of 200 mdeg, a response time of 0.25 s, a scanning speed of 100 nm/min and scanning of three times. The proportion of each secondary structure was fitted and calculated based on Young’s algorithm using a JascoJ-810 spectropolarimeter (Jasco Co., Tokyo, Japan) [27].

### 2.7. Intrinsic Fluorescence Spectroscopy Analysis and Surface Hydrophobicity

Fluorescence spectra of oxidized HDL solution (0.8 mg/mL) were scanned using a spectrofluorometer, where the excitation wavelength was set to 295 nm and the emission wavelength was 300–450 nm, and the changes in fluorescence intensity were recorded. The Δλ was set to 15 and 60 nm, respectively, and the excitation slit width and emission slit width were both 5 nm.

A series of protein solutions at the concentrations of 0.05, 0.10, 0.20, 0.40, 0.60 and 0.8 mg/mL were prepared using 0.01 M, pH 7.4 phosphate buffer. Next, 8-anilino-1-naphthalenesulfonic acid (ANS) (8 mmol/L, 30 μL) as a fluorescent probe was added into 4 mL protein solution, and the mixture was incubated at room temperature for 1 h. The fluorescence intensity of the samples was then measured at the excitation wavelength of 390 nm (slit 5 nm) and the emission wavelength of 470 nm (slit 5 nm) using a fluorescence spectrophotometer (RF-5301pc, Hitachi Co., Tokyo, Japan). The fluorescence intensity was plotted against the protein concentration, and the slope of the curve was used as the surface hydrophobicity index [28].

### 2.8. Determination of Solubility and Turbidity

Oxidized HDL solution (4 mg/mL; pH 8.0) was dissolved in deionized water under magnetic stirring for 30 min, followed by centrifugation at 8000× *g* for 20 min at 4 °C. The supernatant was then collected, and the protein concentration was determined by the Coomassie brilliant blue method. The protein solubility was expressed as the ratio of the concentration in the supernatant to that in the original solution, which was expressed as a percentage [29].

The absorbance of the oxidized HDL solution (4 mg/mL) was measured at 400 nm. The transmittance (%) was then calculated according to the Lambert–Beer’s law (A = −lg*T*). The turbidity (%) of every sample was calculated by the following formula (2).

Turbidity (%) = 100 − *T* (%) (2)

where *T* is the transmittance of sample solution

### 2.9. Particle Size Distribution and Zeta Potential

The particle size distribution and zeta potential of oxidized HDL solutions (1.0 mg/mL) were determined with a previously reported method by the MasterSizer 2000 Nano-ZS instrument (Malvern Instruments Ltd., Worcestershire, UK). In brief, 2 mL (1 mg/mL) of HDL solution was injected into the apparatus, and the results were analyzed using MasterSizer 2000 software [29].

### 2.10. Analysis of Fatty Acid Composition

The stock solution of individual fatty acids was prepared in a fatty-acid-free matrix to obtain a series of fatty acid calibrators at the concentration of 40,000, 20,000, 10,000, 4000, 2000, 1000, 400, 200, 100, 40, 20 or 10 ng/mL. Certain concentrations of decanoic acid-d19, myristic acid-d2, octadecanoic acid-d35, eicosanoic acid-d39 and lignoceric acid-d4 were compounded and mixed as internal standard (IS). The stock solutions of all of these and the working solution were stored in a refrigerator at −20 °C. The samples (100 μL) were taken respectively and homogenized with 300 μL of isopropanol/acetonitrile (1:1) containing mixed IS, and then centrifuged at 12,000 rpm for 10 min. Finally, the supernatant (2 μL) was injected into the LC-MS/MS system for analysis. An ultra-high-performance liquid chromatography coupled to tandem mass spectrometry (UHPLC-MS/MS) system (ExionLC™ AD UHPLC-QTRAP 6500+, AB SCIEX Corp., Boston, MA, USA) was used to quantitate the fatty acids in Novogene Co., Ltd. (Beijing, China). Separation of fatty acids was performed on a Waters ACQUITY UPLC BEH C18 column (2.1 × 100 mm, 1.7 μm), which was maintained at 50 °C. The mobile phase, which consisted of 0.05% formic acid in water (solvent A) and isopropanol/acetonitrile (1:1) (solvent B), was delivered at a flow rate of 0.30 mL/min. The solvent gradient was set as follows: initial 30% B, 1 min; 30–65% B, 2 min; 65–100% B, 11 min; 100% B, 13.5 min; 100–30% B, 14 min; 30% B, 15 min. The mass spectrometer was operated in negative multiple reaction mode (MRM). Parameters were set as follows: IonSpray voltage, −4500 V; curtain gas, 35 psi; ion source temperature, 550 °C; ion source gas of 1 and 2, 60 psi [30].

### 2.11. Statistics Analysis

The obtained experimental data were subjected to statistical analysis using the software SPSS 22.0 (SPSS Inc., Chicago, IL, USA) for one-way ANOVA. All the data expressed as mean ± standard deviation were measured in triplicate. The results were considered statistically significant at *p* < 0.05. PCA analysis and other graphics were conducted using Origin 9.0 (OriginLab Inc., Northampton, MA, USA). The cluster analysis and heatmap were conducted and plotted using Multi Experiment Viewer 4.9 (The Institute for Genomic Research, Annapolis, MD, USA).

## 3. Results and Discussion

### 3.1. SDS-PAGE Analysis of HDL

**Figure 1:**
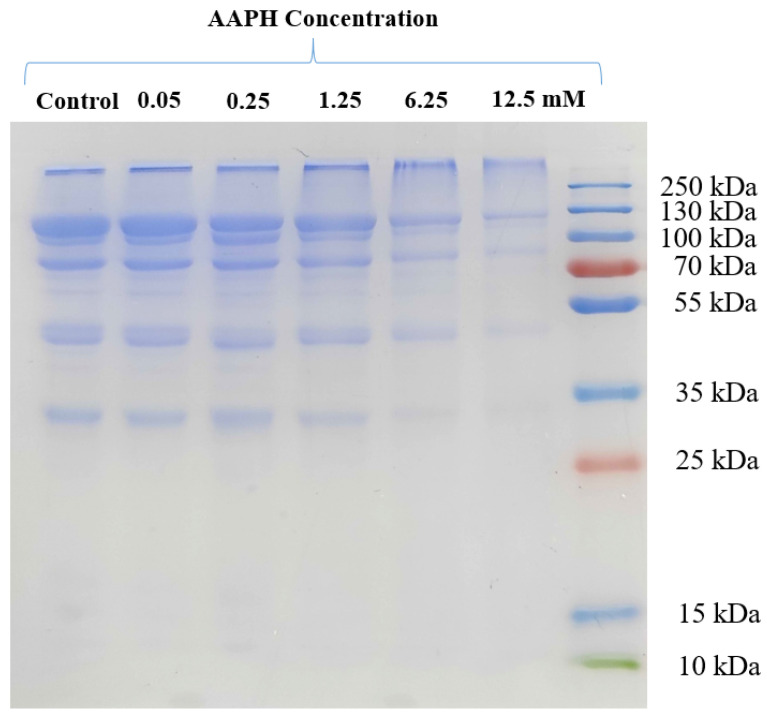
SDS-PAGE profile of HDL treated with different concentrations (0, 0.05, 0.25, 1.25, 6.25, 12.5 mM) of AAPH.

EY-HDL comprised five subunits with the molecular weight of 110, 100, 80, 50 and 35 kDa, respectively. As shown in Figure 1, natural and oxidized HDL mainly showed five subunit bands between 30 and 130 kDa and an integrated molecular band at over 250 kDa. The bands gradually faded with increasing AAPH concentration, probably because AAPH-generated peroxyl radicals led to the oxidative decomposition of HDL subunits into peptides with lower molecular weights (lower than 10 kDa). ROS tend to attack the peptide bonds of proteins to produce fragments, which is initiated by α-hydrogen abstraction to form a carbon-centered radical [31]. A similar previous study has also revealed that the free oxygen radicals derived from AAPH can promote the oxidative decomposition of egg white proteins [32,33]. Moreover, as HDL was oxidized by peroxyl radicals at higher AAPH concentrations (6.25 and 12.5 mM), some hydrophobic groups in the HDL structure were exposed, which would decrease the solubility of HDL in aqueous solution and, thereby, weaken HDL subunit bands.

### 3.2. Carbonyl and Free Sulfhydryl Content of HDL

**Figure 2:**
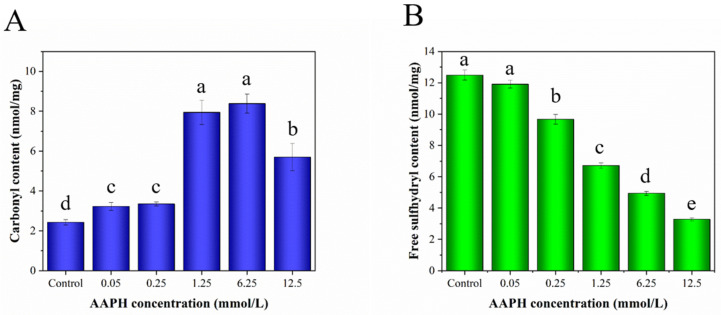
Carbonyl (**A**) and free sulfhydryl content (**B**) of HDL treated with different concentrations of AAPH. Different letters (a, b, c, d, e) indicate that the differences among the samples are significant (*p* < 0.05).

The contents of carbonyls (aldehyde and ketone groups) and free sulfhydryls in proteins are important indicators to evaluate the degree of protein oxidation. Proteins exposed to AAPH will undergo side-chain oxidation and backbone breakage. Protein carbonyl derivatives will be formed from the oxidation of sensitive amino acid residues or the oxidative cleavage of the protein backbone by peroxyl radicals [33,34]. Figure 2A clearly shows that an increase in AAPH concentration promoted the production of carbonyl groups, indicating that the lipid and amino acid side chain on the HDL were oxidized by peroxyl radicals to produce carbonyl groups. AAPH treatment at 0.05 and 0.25 mM resulted in almost equal levels of carbonyl groups, suggesting that peroxyl radicals simultaneously attack the protein backbone and the side chain of α-carbon, which is a direct modification of amino acid side chains by ROS and could produce carbonyls. When the protein backbone was entirely fragmented (peptide bond cleavage), peroxyl radicals mainly led to the formation of carbonyls. Moreover, this process might also involve the adduction of non-protein carbonyl units (lipid-derived carbonyls) [35,36]. Therefore, there was a significant increase in carbonyl groups when the AAPH concentration reached 1.25 mM. There was no significant difference between 1.25 and 6.25 mM AAPH treatment groups, which might be due to a dynamic process of carbonyl group production and conversion into carbon dioxide. The decrease in carbonyl groups at the AAPH concentration of 12.5 mM might be attributed to the further oxidation of numerous carbonyl groups into carbon dioxide. The transformation of sulfhydryl groups into disulfide bonds is partly a free-radical-mediated protein oxidation process. Sulfur-containing amino acid residues (such as cysteine and methionine) are the most vulnerable amino acid residues. Specifically, cysteine can be oxidatively converted into cystine, sulfanic acid, sulfinic acid and sulfonic acid [37]. As shown in Figure 2B, a gradual decrease in the free sulfhydryl groups of HDL occurred with increasing AAPH concentration, suggesting that peroxyl radicals attack the free sulfhydryl groups from methionine and cysteine to generate disulfide bonds or methionyl sulfoxide and sulfenic acid groups, causing the denaturation and aggregation of HDL [38]. A previous study has also demonstrated that methionine, cysteine, tyrosine and tryptophan in meat proteins are the most vulnerable amino acid residues to peroxyl radicals [39].

### 3.3. Fourier-Transform Infrared Spectroscopy (FTIR) and Circular Dichroism (CD) Analysis of HDL

**Figure 3:**
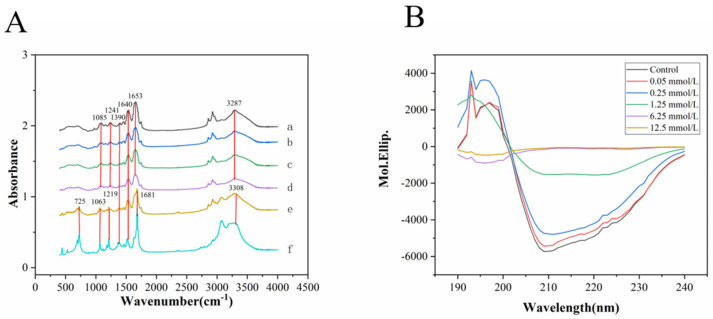
FTIR spectra (**A**) and circular dichroism (**B**) of natural and oxidized HDL. a–f: control, 0.05, 0.25, 1.25, 6.25, 12.5 mM of AAPH.

Generally, protein oxidization will lead to changes in some functional groups. Figure 3A shows that under treatment at 0.05–1.25 mM AAPH, the functional groups in HDL exhibited no significant change, but when the AAPH concentration was increased to 6.25 and 12.5 mM, the peaks of some functional groups showed obvious shifts or increases in intensity. For example, the peaks at 3287 cm^−1^ for groups a–d corresponded to the stretching vibration of hydroxyl groups, while for groups e and f, the peaks shifted to 3308 cm^−1^ (a blue shift), indicating that treatment with AAPH at high concentrations disrupts the intramolecular and intermolecular hydrogen bonds of HDL [40,41]. The characteristic peaks at 1653 cm^−1^ for groups a–d corresponded to the stretching vibration of the amide I band, while these peaks for groups e and f occurred at 1681 cm^−1^, which corresponded to the stretching vibration peak of carbonyl groups, indicating that AAPH at higher concentrations (6.25 and 12.5 mM) alters the secondary structure of HDL. The characteristic peaks at 1640 and 1390 cm^−1^ are assigned to the bending vibration of N–H and the symmetrical stretching vibration of carboxyl groups, respectively. The two absorption bands between 1050 and 1330 cm^−1^ are attributed to the stretching vibration of C–O–C. The characteristic peaks for group e and f at 725 cm^−1^ might be attributed to the rocking vibration of NH_2_, implying that the oxidation of higher concentrations of AAPH would change the vibration type of NH_2_ [42].

Figure 3B clearly shows that natural HDL has a negative acromion at 208 and 222 nm, and a positive peak at 192 nm, which correspond to the α-helical structure. The characteristic peaks at 195, 216 and 200 nm suggested that a β-sheet and a random coil structure are involved in the secondary structure of natural HDL. With increasing treatment concentration of AAPH, the positive peaks at 192 and 195 nm and the negative peaks at 200, 216 and 222 nm showed a downward trend, suggesting that oxidation leads to significant changes in the secondary structure of natural HDL. In addition, Table 1 shows remarkable decreases in α-helical and β-sheet structure with an upward trend of β-turn and random structure, indicating that AAPH treatment tends to transform the secondary structure of natural HDL from an ordered state to a disordered state. In HDL, apolipoprotein is formed by the α-helical structure in its hydrophobic core and the β-sheet structure in hydrophobic amino acids, which are two secondary structures that maintain the stability of the hydrophobic cavity. Therefore, the decrease in α-helix and β-sheet structure can easily lead to changes in the cavity structure and, thus, break the interaction between protein and lipid molecules [24,43]. For example, the treatment of rice proteins with lipid peroxidation primary products led to the formation of protein carbonyls and loss of sulfhydryls, which was accompanied by the breakage of secondary structure (α-helix, β-turn and random coil) and rise of β-sheet content [44].

### 3.4. Analysis of Endogenous Fluorescence and Surface Hydrophobicity

**Figure 4:**
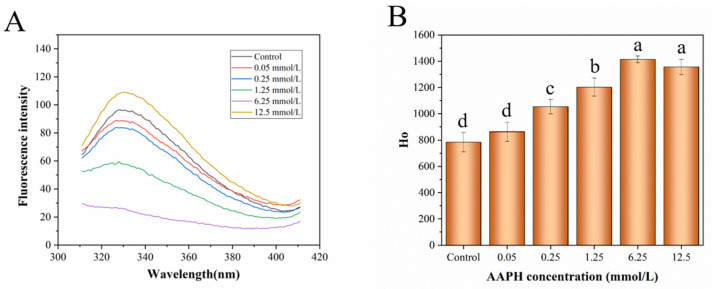
Endogenous fluorescence analysis (**A**) and surface hydrophobicity (**B**) of natural and oxidized HDL. Different letters (a, b, c, d) indicate that the differences among the samples are significant (*p* < 0.05).

Proteins can show different fluorescence peaks due to the different side-chain chromophores of Trp, Tyr and Phe. Among them, Phe has the lowest fluorescence intensity and can help to transfer energy from the Tyr residue to the Trp residue, resulting in fluorescence quenching of the Tyr residue. Therefore, Trp is often used as an endogenous probe to study the changes in the microenvironment of protein and protein folding kinetics, so as to infer the changes in the tertiary structure of protein [45,46]. The Trp residue can produce fluorescence intensity at an excitation wavelength of 295 nm and an emission wavelength range of 300–400 nm. Endogenous fluorescence can be used to evaluate the effect of an oxidant on the Trp residue of HDL. Free radicals can transform Trp residues with low single-electron oxidation potential into an unstable state (such as hydroperoxide and alcohol), thereby decreasing the endogenous fluorescence intensity [47,48]. As shown in Figure 4A, with increasing AAPH concentration, the endogenous fluorescence intensity of HDL decreased significantly, indicating that Trp residues in HDL were converted into other products by peroxyl radicals. The fluorescence intensity of HDL increased under treatment by AAPH at a concentration of 12.5 mM, indicating that, with the proceeding of oxidation reaction, protein aggregation might return the Trp residues to the nonpolar environment of HDL [49].

The fluorescence intensity increasing linearly with the growth of protein concentration can accurately and indirectly reflect the hydrophobicity of proteins. Figure 4B presents the surface hydrophobicity calculated based on the fluorescence intensity of different concentrations of natural and oxidized HDL. The hydrophobicity of HDL showed a notable rising trend after oxidization by different concentrations of AAPH, suggesting that free radicals generated from AAPH broke the advanced structure of HDL and exposed the hydrophobic amino residues (such as Tyr and Trp) from the intrinsic hydrophobic region, which also implies that HDL experiences an aggregation process through hydrophobic interaction [50].

### 3.5. Solubility and Turbidity Measurements

**Figure 5:**
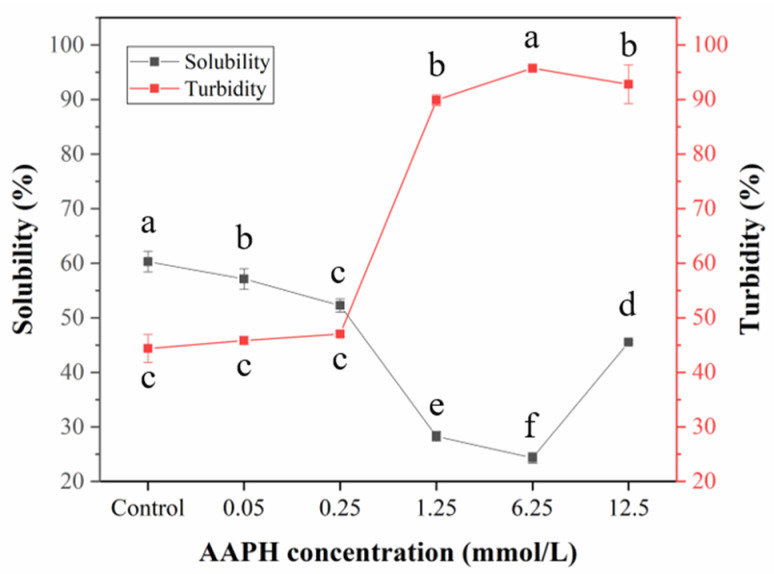
Solubility and turbidity of natural and oxidized HDL solution. The black line and red line reflect the solubility and turbidity of the samples, respectively. Different letters (a, b, c, d, e, f) indicate that the differences among the samples are significant (*p* < 0.05).

Protein oxidation can result in the exposure of hydrophobic groups from the internal structure, and some aggregates might be formed due to the hydrophobic interactions of protein molecules, which negatively affects the solubility of protein [51,52]. Figure 5 clearly shows that the solubility of HDL decreased gradually from about 60% to 20% with the AAPH concentration increasing from 0.05 to 6.25 mM, indicating that some insoluble aggregates were formed after the oxidation of HDL. The obvious aggregation including disulfide bonds and non-disulfide interaction between protein subunits would also result in a decrease in solubility, which is generally in agreement with the trend of surface hydrophobicity. The mixture of whey protein isolate with oxidizing unsaturated lipids has been demonstrated to form heterogeneous aggregates dominated by disulfide bonds [53]. The significant decline of protein solubility caused by aggregation is generally a dominant factor for the loss of protein functionality. Interestingly, when the concentration of AAPH reached 12.5 mM, the solubility showed a significant increase, which might be due to the formation of some hydrophilic compounds after the oxidative degradation of HDL. Turbidity is an important indicator for the aggregation degree of protein under different treatment conditions. The turbidity of HDL solution showed an obvious rise from around 45% to 90% after oxidation, suggesting the formation of more insoluble HDL aggregates. Due to their electrophilic property, protein carbonyls generated in protein-rich food products have the potential to interact with amine and sulfur groups to produce protein aggregates [54]. Furthermore, a study of soy proteins treated by a model oxidation system comprising lipoxygenase and linoleic acid indicated the formation of disulfide and non-disulfide bonds, which play a vital role in protein aggregation [55].

### 3.6. Characterization of Particle Size and Zeta Potential

**Figure 6:**
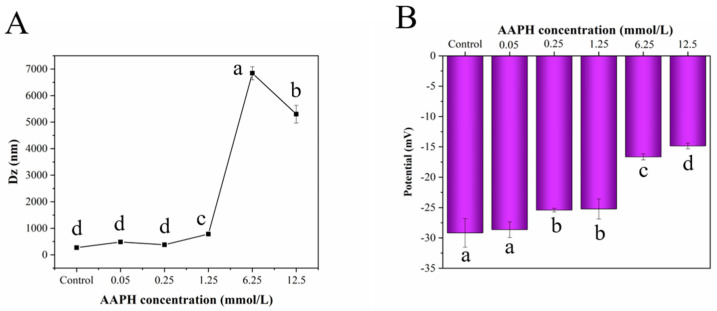
Particle size (**A**) and zeta potential (**B**) of natural and oxidized HDL solution. Different letters (a, b, c, d) indicate that the differences among the samples are significant (*p* < 0.05).

Dynamic light scattering is an effective technique to monitor the formation of protein aggregates. AAPH-induced oxidation could cause the aggregation of protein in the aqueous solution, thus changing the protein’s particle size distribution. As shown in Figure 6A, the particle size of the HDL solution exhibited a slight increase (from around 250 to 750 nm) after treatment with 0.05–1.25 mM AAPH, probably because AAPH-generated free radicals induced the covalent crosslinking among protein molecules or their subunits and the exposure of hydrophobic groups [56]. When the concentration of AAPH was increased to 6.25 mM, the particle size was sharply increased to around 6.5 μm, indicating the occurrence of intense aggregation during protein oxidation. However, the particle size decreased when the AAPH concentration was further increased to 12.5 mM because excessive oxidation promoted the disaggregation of some protein aggregates, which were re-dissolved in the aqueous solution. These results are in accordance with the data of surface hydrophobicity and solubility. Zeta potential is usually used to reflect the surface charge of the solution comprising different saline ions or molecules and is a vital indicator to evaluate the solution stability. A higher absolute value of the zeta potential implies a higher stability of the solution system. Some molecules in the solution will be aggregated or assembled with decreasing solution stability [57]. As depicted in Figure 6B, the absolute zeta potential of HDL solution decreased notably with increasing AAPH concentration, suggesting that the oxidized HDL solution was prone to be unstable. These results imply that the side chain of amino acid residues was oxidized, changing the degree of protein molecule protonation and reducing the polarity of protein, thereby declining the water-holding capacity of the protein [22]. Therefore, both covalent cross-linking and non-covalent interactions can lead to extensive aggregation and decline the solubility of proteins.

### 3.7. Analysis of Fatty Acid Composition

HDL from egg yolk is constituted by 75–80% proteins and 20–25% lipids (phospholipids, triglycerides and cholesterol), among which lipids, particularly fatty acids, are sensitive to oxidization by free radicals. Spherical HDL is an apolipoprotein with a highly ordered structure composed of the α-helical structure of its hydrophobic core and β-sheet structure of hydrophobic amino acids, which jointly maintain the structural stability of HDL [43]. The hydrophobic cavity of HDL can be easily exposed when the ordered secondary structure is disrupted. As a result, the phospholipid molecules embedded in the cavity easily lose their protection and are dissociated to be oxidatively decomposed [7]. In this study, the fatty acid composition of natural and oxidized HDL was analyzed by LC-MS and displayed in Table 2. Clearly, a total of 39 types of fatty acids were found in natural HDL. The levels of saturated, mono-unsaturated, poly-unsaturated and total fatty acids all showed significant decreases after the oxidization of HDL by low concentrations (0.05–1.25 mM) of AAPH, while they exhibited an upward trend when the concentration of AAPH was increased from 1.25 to 12.5 mM. AAPH-derived peroxyl radicals tend to react with fatty acids to cause their auto-oxidation and degradation for forming some volatile products such as aldehyde and ketone, thus reducing the level of fatty acids [58]. Moreover, some long-chain fatty acids could be oxidized to produce short-chain fatty acids, which contributes to the increase in total fatty acids. More specifically, the levels of some unsaturated fatty acids, including heptadecenoic acid (C17:1), elaidic acid (C18:1), vaccenic acid (C18:1), oleic acid (C18:1), linoleic acid (C18:2), gamma-linolenic acid (C18:3), nonadecenoic acid (C19:1), eicosapentaenoic acid (C20:5), docosadienoic acid (C22:2) and docosapentaenoic acid (C22:5), steadily decreased due to the oxidation of carbon–carbon double bonds with gradually increasing AAPH concentration. On the contrary, the content of tetradecanoic acid (C14:0) and palmitelaidic acid (C16:1) showed continuous increases with increasing AAPH concentration. These results suggest that a large number of longer-chain fatty acids are oxidatively broken into shorter-chain fatty acids. Additionally, peroxyl radicals could attack fatty acids in HDL to form alkyl radicals, which will promote the chain proliferation in fatty acid oxidation [59]. Accordingly, some oxidation products from fatty acids contribute to the oxidation of amino acid residues of protein [60]. As a result, the molecular structure of HDL is continuously destroyed, and some physicochemical properties are negatively affected.

**Figure 7:**
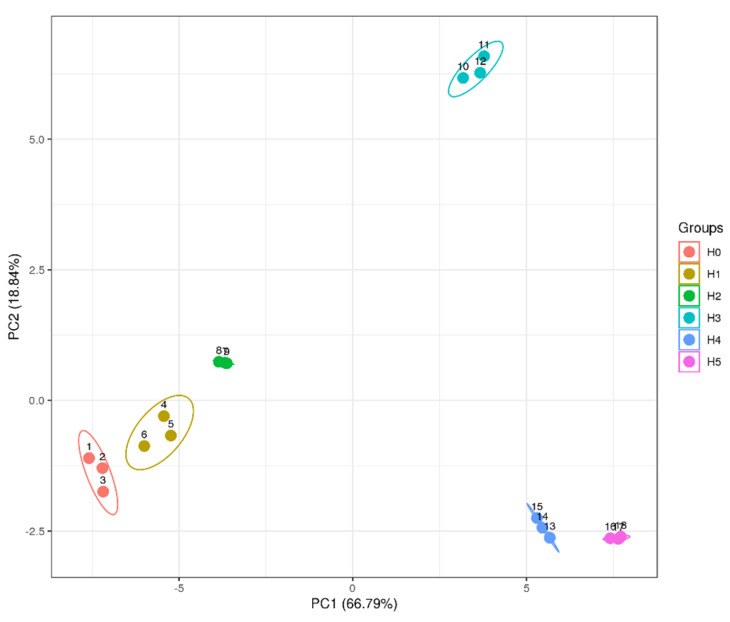
PCA analysis of fatty acids from natural and oxidized HDL. H_0_ represents natural HDL, and H_1_ to H_5_ represent natural HDL oxidized by different concentrations of AAPH. Principal component 1 (PC1) and principal component 2 (PC2), respectively, account for 66.79% and 18.84% of the variation in the PCA plot.

**Figure 8:**
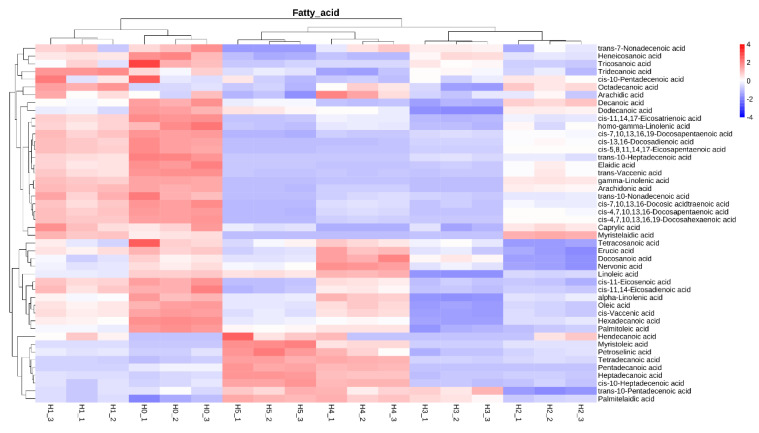
Cluster analysis of fatty acids from natural and oxidized HDL. H_0_ represents natural HDL, and H_1_ to H_5_ represent natural HDL oxidized by different concentrations of AAPH.

As shown in Figure 7, the PCA plot composed of PC1 (66.79%) and PC2 (18.84%) could distinctly distinguish the principal components of fatty acids in natural and oxidized HDL, indicating that AAPH oxidization can greatly change the types of fatty acids in HDL. According to the PCA results, there were six well-defined groups (H_0_ to H_5_). PC1 has a positive value for H_3_, H_4_ and H_5_ groups and a negative value for H_0_, H_1_ and H_2_ groups. As for PC2, the H_0_, H_1_, H_4_ and H_5_ groups are closer to each other and have a negative value, and only the H_2_ and H_3_ groups possess a positive value. Combined with the results in Figure 8, it can be inferred that PC1 is mainly affected by caprylic acid, myristelaidic acid, linoleinic acid, eicosenoic acid, oleic acid, vaccenic acid, palmitoleic acid, hendecanoic acid, myristoleic acid, petroselinic acid, tetradecanoic acid, heptadecanoic acid, heptadecenoic acid, pentadecenoic acid and pamitelaidic acid. Additionally, PC2 is mainly influenced by heneicosanoic acid, tridecanoic acid, archidic acid, decanoic acid, caprylic acid, decosanoic acid, nervonic acid, linoleic acid, hendecanoic acid, tretradecanoic acid, pentadecanoic acid, heptadecanoic acid and heptadecenoic acid. The PCA and cluster analysis of fatty acids further demonstrated the differences in the content and type of fatty acids between natural and oxidized HDL.

## 4. Conclusions

An AAPH-derived peroxyl radical system was used as a trigger to evaluate the oxidative effect of radicals on the secondary/tertiary structure, physicochemical properties and fatty acid composition of egg yolk HDL. When natural HDL was treated with different concentrations of AAPH, the secondary/tertiary structure and relative functional groups showed significant variations. The hydration property and dispersibility of HDL powder were negatively affected as a result of the oxidative aggregation of protein. Notably, the level and type of fatty acids in the three-dimensional structure of HDL varied remarkably. Research on the oxidation of egg yolk HDL will be helpful to elucidate the molecular mechanism for egg yolk oxidation.

## Figures and Tables

**Table 1:** Secondary structure content of natural and oxidized HDL.

Secondary Structure	Control	0.05 mM	0.25 mM	1.25 mM	6.25 mM	12.5 mM
α-Helix	29.40 ± 1.20	21.70 ± 0.85	23.20 ± 0.85	24.05 ± 0.89	26.70 ± 0.28	27.30 ± 0.90
β-Sheet	31.45 ± 2.51	33.9 ± 2.40	23.15 ± 2.26	21.38 ± 3.46	14.50 ± 1.34	12.80 ± 1.23
β-Turn	13.73 ± 1.10	18.25 ± 0.53	23.65 ± 2.05	24.43 ± 6.40	26.33 ± 2.56	26.70 ± 2.78
Random	25.43 ± 0.28	26.15 ± 0.99	30.00 ± 1.06	30.20 ± 1.03	32.50 ± 0.25	33.20 ± 0.44

**Table 2:** Fatty acid composition of natural and oxidized HDL.

Fatty Acid (μg/g)	H_0_	H_1_	H_2_	H_3_	H_4_	H_5_
Caprylic acid (C8:0)	2.33 ± 0.487 ^a^	2.56 ± 0.487 ^a^	1.90 ± 0.128 ^b^	0.68 ± 0.390 ^d^	0.24 ± 0.024 ^e^	1.06 ± 0.084 ^c^
Decanoic acid (C10:0)	0.41 ± 0.022 ^a^	0.24 ± 0.089 ^c^	0.32 ± 0.015 ^b^	0.09 ± 0.013 ^e^	0.13 ± 0.006 ^d^	0.22 ± 0.009 ^c^
Dodecanoic acid (C12:0)	0.39 ± 0.019 ^a^	0.20 ± 0.017 ^d^	0.27 ± 0.005 ^b,c^	0.05 ± 0.004 ^e^	0.23 ± 0.017 ^c,d^	0.27 ± 0.013 ^d^
Tetradecanoic acid (C14:0)	2.98 ± 0.290 ^c^	3.49 ± 0.16 ^b^	3.80 ± 0.090 ^b^	3.50 ± 0.104 ^b^	8.63 ± 0.175 ^a^	9.11 ± 0.28 ^a^
Myristelaidic acid (C14:1, n-9, cis)	2.56 ± 0.140 ^c^	3.62 ± 0.202 ^b^	4.12 ± 0.101 ^a^	0.104 ± 0.002 ^d^	ND	ND
Pentadecanoic acid (C15:0)	0.81 ± 0.061 ^c^	0.431 ± 0.016 ^d^	0.229 ± 0.024 ^e^	0.226 ± 0.010 ^e^	1.97 ± 0.031 ^b^	2.30 ± 0.1130 ^a^
Pentadecenoic acid (C15:1, n-10)	0.159 ± 0.015 ^b^	0.15 ± 0.010 ^b^	0.08 ± 0.006 ^c^	0.225 ± 0.019 ^a^	0.22 ± 0.020 ^a^	0.235 ± 0.016 ^a^
Hexadecanoic acid (C16:00)	118.78 ± 1.052 ^a^	77.15 ± 2.55 ^b^	5.97 ± 1.954 ^c^	34.63 ± 0.354 ^d^	73.23 ± 2.31 ^b^	65.50 ± 1.65 ^e^
Palmitoleic acid (C16:1, n-9, trans)	6.71 ± 0.166 ^a^	3.29 ± 0.161 ^b^	1.98 ± 0.209 ^c^	1.07 ± 0.406 ^d^	4.45 ± 38.62 ^e^	3.79 ± 0.034 ^f^
Palmitelaidic acid (C16:1, n-9, cis)	2.57 ± 0.522 ^a^	3.59 ± 0.216 ^b^	3.54 ± 0.146 ^b^	4.97 ± 0.242 ^c^	5.77 ± 0.289 ^d^	6.04 ± 0.133 ^d^
Heptadecanoic acid (C17:0)	0.99 ± 0.091 ^a^	110 ± 0.039 ^a^	0.85 ± 0.026 ^b^	0.74 ± 0.060 ^b^	2.36 ± 0.041 ^c^	2.93 ± 0.018 ^d^
Heptadecenoic acid (C17:1, n-10, cis)	0.91 ± 0.207 ^a^	0.88 ± 0.221 ^a^	0.59 ± 0.150 ^a,b^	0.51 ± 0.090 ^b^	3.11 ± 0.015 ^c^	3.54 ± 0.148 ^d^
Heptadecenoic acid (C17:1, n-10, trans)	1.81 ± 0.083 ^a^	1.03 ± 0.068 ^b^	0.46 ± 0.058 ^c^	0.374 ± 0.033 ^c^	ND	ND
Octadecanoic acid (C18:0)	18.45 ± 0.257 ^c^	23.84 ± 0.457 ^a^	21.56 ± 0.498 ^b^	16.67 ± 1.178 ^d^	20.956 ± 0.772 ^b^	17.51 ± 0.544 ^c,d^
Oleic acid (C18:1, n-9)	157.73 ± 5.79 ^a^	118.81 ± 4.478 ^b^	82.32 ± 3.910 ^c^	52.86 ± 2.183 ^d^	129.72 ± 5.49 ^e^	92.720 ± 0.928 ^f^
Vaccenic acid (C18:1, n-9, cis)	217.58 ± 3.204 ^a^	163.61 ± 5.307 ^b^	113.88 ± 6.446 ^c^	71.40 ± 1.641 ^d^	171.81 ± 5.77 ^b^	123.503 ± 4.73 ^c^
Elaidic acid (C18:1, n-9, cis)	28.19 ± 1.479 ^a^	17.79 ± 0.999 ^b^	14.36 ± 1.099 ^c^	7.11 ± 0.271 ^d^	6.43 ± 0.086 ^d^	6.948 ± 0.244 ^d^
Vaccenic acid (C18:1, n-9, trans)	0.03 ± 0.328 ^a^	18.60 ± 1.388 ^b^	14.72 ± 1.514 ^c^	7.11 ± 0.241 ^d^	7.07 ± 0.393 ^d^	7.08 ± 0.269 ^d^
Petroselinic acid (C18:1, n-6)	0.73 ± 0.097 ^a,b^	0.91 ± 0.034 ^a^	0.81 ± 0.061 ^a^	0.50 ± 0.092 ^b^	1.50 ± 0.266 ^c^	2.193 ± 0.124 ^d^
Linoleic acid (C18:2, n-6, cis)	21.71 ± 0.354 ^a^	13.80 ± 0.315 ^b^	11.16 ± 0.315 ^c^	3.49 ± 0.213 ^d^	2.48 ± 0.446 ^e^	19.22 ± 0.558 ^f^
gamma-Linolenic acid (C18:3, n-3, cis)	8.23 ± 0.117 ^a^	6.45 ± 0.412 ^b^	4.91 ± 0.148 ^c^	0.27 ± 0.004 ^d^	0.65 ± 0.015 ^d^	0.392 ± 0.012 ^d^
alpha-Linolenic acid (C18:3, n-3, cis)	0.99 ± 0.069 ^a^	0.605 ± 0.047 ^b^	0.463 ± 0.027 ^c^	0.081 ± 0.34 ^d^	0.89 ± 0.050 ^e^	0.489 ± 0.008 ^c^
Nonadecenoic acid (C19:1, n-10, trans)	0.617 ± 0.096 ^a^	0.54 ± 0.067 ^a^	0.22 ± 0.003 ^b^	0.13 ± 0.013 ^b,c^	0.15 ± 0.027 ^b,c^	0.07 ± 0.003 ^c^
Eicosenoic acid (C20:1, n-11, cis)	18.49 ± 0.519 ^a^	16.26 ± 0.451 ^b^	11.02 ± 0.580 ^d^	11.32 ± 0.386 ^d^	15.32 ± 0.239 ^c^	11.02 ± 0.060 ^d^
Arachidic acid (C20:0)	2.31 ± 0.531 ^a,b^	2.67 ± 0.408 ^a,c^	1.95 ± 0.289 ^a,d^	1.72 ± 1.66 ^a,d^	3.15 ± 0.409 ^c^	1.27 ± 0.258 ^d^
Eicosadienoic acid (C20:2, n-11, cis)	3.29 ± 0.230 ^a^	2.80 ± 0.194	1.69 ± 0.042 ^c^	1.59 ± 0.052 ^c^	2.42 ± 0.071 ^d^	1.53 ± 0.091 ^c^
Arachidonic acid (C20:4)	185.14 ± 3.364 ^a^	165.10 ± 3.728 ^b^	110.46 ± 2.710 ^c^	29.73 ± 0.923 ^d^	48.85 ± 0.817 ^e^	28.86 ± 0.823 ^d^
Eicosatrienoic acid (C20:3, n-11, cis)	6.48 ± 0.111 ^a^	4.642 ± 0.192 ^b^	2.95 ± 0.136 ^c^	1.639 ± 0.035 ^d^	3.13 ± 0.014 ^c^	1.996 ± 0.043 ^e^
Eicosapentaenoic acid (C20:5, n-8, cis)	14.50 ± 1.281 ^a^	10.67 ± 0.510 ^b^	6.052 ± 0.158 ^c^	1.37 ± 0.056 ^d^	0.553 ± 0.010 ^d^	0.251 ± 0.008 ^d^
Heneicosanoic acid (C21:0)	1.93 ± 0.184 ^a^	1.28 ± 0.020 ^b^	0.853 ± 0.023 ^c^	1.28 ± 0.112 ^b^	0.498 ± 0.036 ^d^	0.418 ± 0.072 ^d^
Docosanoic acid (C22:0)	8.11 ± 0.433 ^a^	6.37 ± 0.500 ^b^	4.21 ± 0.212 ^c^	7.63 ± 0.291 ^a^	10.29 ± 0.492 ^d^	6.45 ± 0.090 ^b^
Erucic acid (C22:1, n-13)	2.126 ± 0.081 ^a^	1.909 ± 0.056 ^b^	1.13 ± 0.088 ^c^	1.55 ± 0.101 ^d^	2.23 ± 0.085 ^a^	1.53 ± 0.054 ^d^
Docosadienoic acid (C22:2, n-13, cis)	2.00 ± 0.124 ^a^	1.49 ± 0.085 ^b^	0.912 ± 0.038 ^c^	0.43 ± 0.008 ^d^	0.221 ± 0.036 ^e^	0.18 ± 0.010 ^e^
Docosic acid traenoic acid (C22:4)	22.93 ± 0.850 ^a^	17.58 ± 1.088 ^b^	11.439 ± 0.350 ^c^	7.50 ± 0.354 ^d^	9.88 ± 0.347 ^e^	6.15 ± 0.054 ^f^
Docosapentaenoic acid (C22:5, n-7, cis)	11.17 ± 0.240 ^a^	8.76 ± 0.616 ^b^	5.293 ± 0.158 ^c^	3.434 ± 0.099 ^d^	2.69 ± 0.175 ^e^	1.29 ± 0.085 ^f^
Docosapentaenoic acid (C22:5, n-7, cis)	89.89 ± 0.310 ^a^	72.55 ± 3.176 ^b^	48.19 ± 1.436 ^c^	23.28 ± 0.196 ^d^	33.28 ± 0.463 ^e^	19.03 ± 0.588 ^f^
Docosahexaenoic acid (C22:6, n-4, cis)	41.465 ± 0.911 ^a^	32.299 ± 0.992 ^b^	20.95 ± 0.390 ^c^	8.58 ± 0.123 ^d^	10.24 ± 0.159 ^e^	5.39 ± 0.062 ^f^
Tetracosanoic acid (C24:0)	0.73 ± 0.068 ^a^	0.60 ± 0.032 ^b^	0.50 ± 0.006 ^c^	0.61 ± 0.012 ^b^	0.67 ± 0.016 ^a,b^	0.610 ± 0.026 ^b^
Nervonic acid (C24:1, n-15)	4.31 ± 0.129 ^a^	3.354 ± 0.125 ^b^	1.88 ± 0.160 ^c^	3.32 ± 0.121 ^b^	5.68 ± 0.140 ^d^	3.66 ± 0.126 ^e^
SFA	158.20 ± 3.497 ^a^	119.93 ± 4.703 ^b,c^	96.137 ± 3.271 ^c^	67.82 ± 2.693 ^d^	122.35 ± 4.328 ^b^	107.65 ± 3.160 ^c^
MUFA	472.00 ± 12.862 ^a^	354.33 ± 13.782 ^b^	251.12 ± 14.532 ^c^	162.54 ± 5.842 ^d^	353.45 ± 12.861 ^b^	262.33 ± 5.902 ^c^
PUFA	407.80 ± 7.960 ^a^	336.75 ± 11.354 ^b^	224.45 ± 5.908 ^c^	81.39 ± 2.064 ^e^	137.64 ± 2.602 ^d^	84.77 ± 2.344 ^e^
Total FA	1038.01 ± 24.320 ^a^	811.01 ± 29.839 ^b^	571.72 ± 23.711 ^c^	311.75 ± 10.600 ^e^	613.44 ± 19.791 ^c^	454.75 ± 11.407 ^d^

Notes: H_0_ represents natural HDL; H_1_ to H_5_ represent natural HDL oxidized by different concentrations of AAPH. Different letters (a, b, c, d, e and f) at the same row indicate a significant difference (*p* < 0.05).

## Data Availability

The data presented in this study are available on request from the corresponding author. The data are not publicly available due to the privacy.
